# Arginines of the CGN codon family are Achilles’ heels of cancer genes

**DOI:** 10.1038/s41598-024-62553-7

**Published:** 2024-05-22

**Authors:** Mária Trexler, László Bányai, Krisztina Kerekes, László Patthy

**Affiliations:** Institute of Enzymology, HUN-REN Research Centre for Natural Sciences, Budapest, 1117 Hungary

**Keywords:** Biochemistry, Cancer, Evolution, Genetics, Molecular biology, Structural biology, Diseases, Medical research, Molecular medicine, Oncology, Pathogenesis

## Abstract

Recent studies have revealed that arginine is the most favorable target of amino acid alteration in most cancer types and it has been suggested that the high preference for arginine mutations reflects the critical roles of this amino acid in the function of proteins. High rates of mutations of arginine residues in cancer, however, might also be due to increased mutability of arginine codons of the CGN family as the CpG dinucleotides of these codons may be methylated. In the present work we have analyzed spectra of single base substitutions of cancer genes (oncogenes, tumor suppressor genes) and passenger genes in cancer tissues to assess the contributions of CpG hypermutability and selection to arginine mutations. Our studies have shown that arginines encoded by the CGN codon family display higher rates of mutation in both cancer genes and passenger genes than arginine codons AGA and AGG that are devoid of CpG dinucleotide, suggesting that the predominance of arginine mutations in cancer is primarily due to CpG hypermutability, rather than selection for arginine replacement. Nevertheless, our results also suggest that CGN codons for arginines may serve as Achilles' heels of cancer genes. CpG hypermutability of key arginines of proto-oncogenes, leading to high rates of recurrence of driver mutations, contributes significantly to carcinogenesis. Similarly, our results indicate that hypermutability of the CpG dinucleotide of CGA codons (converting them to TGA stop codons) contributes significantly to recurrent truncation and inactivation of tumor suppressor genes.

## Introduction

Recent analyses of genome-wide mutational spectra have revealed that arginine is the most favorable target of amino acid alteration in most cancer types studied^1^. Analyses of the amino acid mutation patterns of cancer have also shown that the Arg → His substitution is dominant followed by Arg → Gln and Arg → Cys substitutions^2,3^. The authors of these studies assumed that the high preference of mutations to affect arginines in cancer is related to the unique properties of this amino acid residue that endow it with major physiological importance. For example, it has been suggested that this preference reflects the fact that arginine plays a pivotal role in cellular physiology and is intimately involved with cell signaling related to tissue repair processes^1^. Anoosha et al.^2^ suggested that the high preference of Arg might be due to the fact that it is the most favored residue for binding with DNA, can form multiple hydrogen bonds and plays an important role as stabilizing element in proteins. It has also been pointed out that since the Arg → His mutations swap a positively charged amino acid for a titratable amino acid these mutations can confer pH sensitivity to the mutant protein and alter its function^3,4^.

It must be pointed out, however, that arginine residues of proteins have another unique aspect: four of the six codons encoding arginine belong to the CGN codon family and methylation of the CpG dinucleotide present in these codons would result in hypermutability of these codons. Indeed, in our previous study we have shown that methylation and hypermutability of the CpG dinucleotides of the CGA codons for arginine explains why TGA is used at a much higher frequency than TAG as stop codons of vertebrate proteins^5^. Thus, an alternative explanation for the high rates of mutations of arginine residues in cancer is that it reflects high mutability of arginine codons, rather than selection for the replacement of arginines by other residues. In the present work we have analyzed single base substitutions of cancer genes (oncogenes, tumor suppressor genes) and passenger genes in cancer tissues to assess the contribution of this factor to the amino acid mutation patterns of cancer.

In the present work we have shown that arginines encoded by the CGN codon family display high rates of mutation in both cancer genes and passenger genes due to methylation and hypermutability of their CpG sites, resulting in Arg → His, Arg → Gln, Arg → Cys, Arg → Trp and Arg → Stop mutations. It is noteworthy that mutation rates of arginine codons AGA and AGG (devoid of CpG dinucleotide) were significantly lower, confirming that the predominance of arginine mutations in cancer is primarily due to CpG hypermutability, rather than selection for arginine replacement.

Nevertheless, our results also suggest that CGN codons for arginines may serve as Achilles' heels of cancer genes. We have shown that CpG hypermutability of key arginines of proto-oncogenes (leading to high rates of recurrence of driver mutations), contributes significantly to carcinogenesis. Similarly, our results indicate that hypermutability of the CpG dinucleotide of the CGA codon (converting it to a TGA stop codon) contributes significantly to recurrent truncation and inactivation of tumor suppressor genes.

## Results and discussion 

### Impact of methylation and hypermutability of CpG dinucleotides on amino acid mutation spectra of human genes in cancer

There are eight codons that contain CpG dinucleotides (Supplementary Table 1). As shown in this table, methylation of cytosines of CpG dinucleotides of these codons in the sense strand is expected to favor CG > TG changes resulting in seven missense and one nonsense mutation: (ACG (Thr) > ATG (Met), CCG (Pro) > CTG (Leu), CGA (Arg) > TGA (STOP), CGC (Arg) > TGC (Cys), CGG (Arg) > TGG (Trp), CGT (Arg) > TGT (Cys), GCG (Ala) > GTG (Val) and TCG (Ser) > TTG (Leu). On the other hand, methylation of cytosines of CpG dinucleotides of these codons in the antisense strand is expected to lead to silent substitutions of Thr (ACG > ACA), Pro (CCG > CCA), Ala (GCG > GCA) and Ser (TCG > TCA), whereas substitutions of the four CGN (Arg) codons lead to missense mutations (CGA > CAA, Arg > Gln; CGC > CAC, Arg > His; CGG > CAG, Arg > Gln; CGT > CAT, Arg > His). In other words, the four Arg codons of the CGN codon family are special among CpG containing codons in that C > T mutation of CpG of both the sense and antisense strand result in a change of the amino acid, whereas in the case of the NCG codons of Thr, Pro, Ala and Ser only the sense strand mutations alter the amino acid. It has been pointed out earlier that this difference of CpG bearing codons has significant evolutionary consequences for organisms with methylated genomes^6^. Thanks to purifying selection, NCA codons for Thr, Pro, Ala and Ser increase preferentially with stronger methylation, indicating that depression of NCG codons in strongly methylated genes occurs through silent C > T substitutions on the antisense strand. The CGN group of CpG bearing codons, which code for arginine evolve differently because C > T substitutions on both the sense and antisense stands produce amino acid changes. The authors have shown that arginine content is negatively correlated with gene body methylation, suggesting that the shift in arginine content is due to CpG hypermutability of these codons^6^.

To permit the distinction of the effects of hypermutability and selection on somatic amino acid mutation spectra, we have analyzed three categories of human genes: selectively neutral passenger genes; proto-oncogenes positively selected for key missense mutations but negatively selected against nonsense mutations; tumor suppressor genes, positively selected for missense and nonsense mutations^7^. In these studies we have used the lists of oncogenes (OGs) and tumor suppressor genes (TSGs) defined by Vogelstein et al.^8^ and the list of passenger genes (PGs) we have characterized earlier^7,9^. To analyze the impact of CpG methylation on mutation spectra we have compared the relative frequency of C > T (G > A) substitutions of nucleotides present in CpG dinucleotides (abbreviated as CSS, C > T substitution of CpG sites, sense strand; CAS, C > T substitution of CpG sites, antisense strand; 3rdC, C > T substitution of CpG sites spanning codon boundaries, sense strand; 1stG, C > T substitution of CpG sites spanning codon boundaries, antisense strand) with those of C (G) nucleotides that are not part of CpG dinucleotide sequences (abbreviated as RESTS, C > T substitution of sites that are not part of CpG dinucleotides, sense strand; RESTA, C > T substitution of sites that are not part of CpG dinucleotides, antisense strand). For each C (G) site of the various codons we have determined the fraction of sites (fN) that experienced a C > T (G > A) substitution at least once. The fN value was multiplied by the number of times the mutation was observed to calculate fN*, the parameter reflecting recurrence of that substitution.

#### Passenger genes

**Figure 1:**
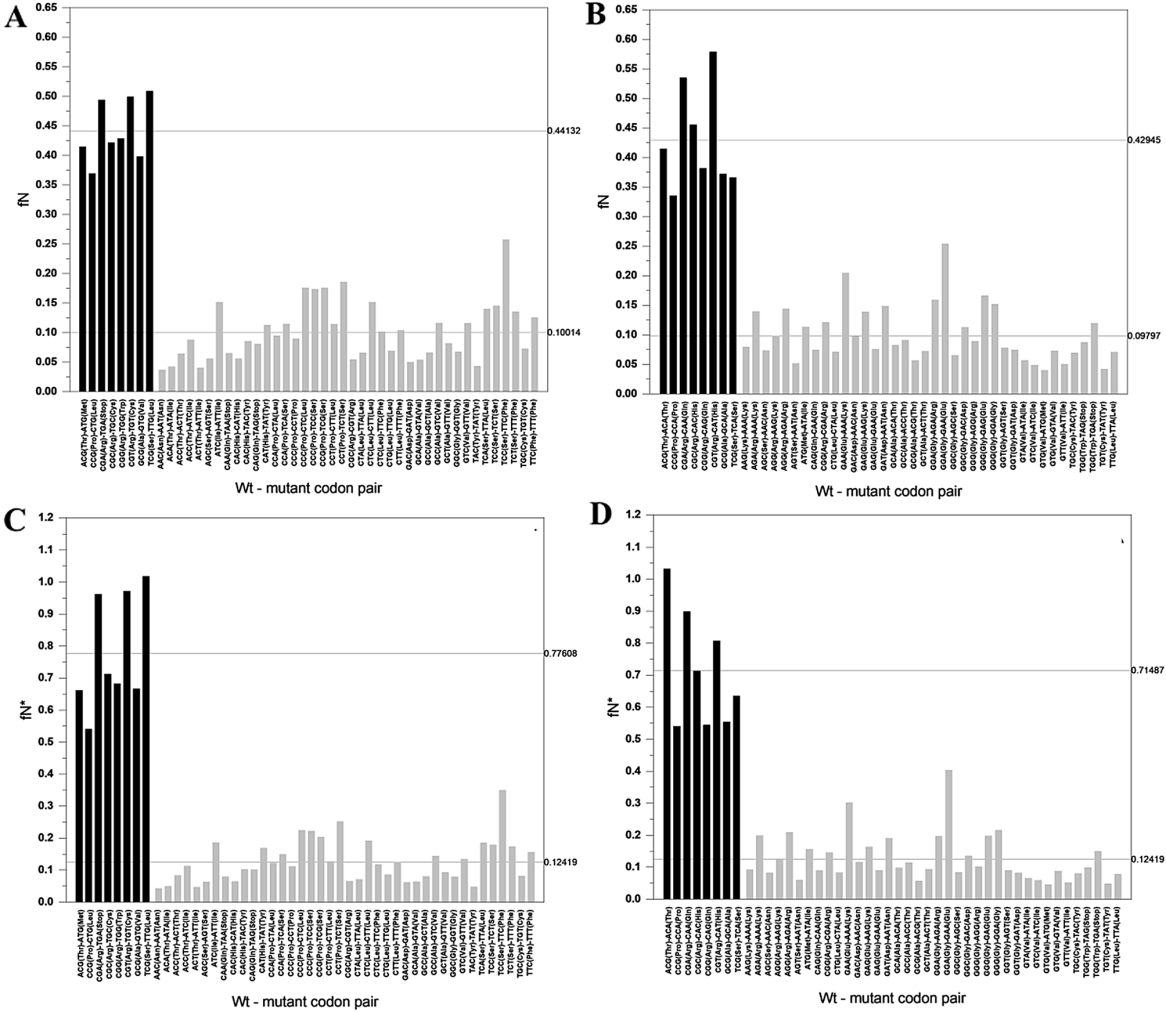
Frequency and recurrence of somatic C > T and G > A substitutions of passenger genes (PGs). The abscissas list the wild type and mutant codon pairs resulting from C > T (**A,C**) or G > A (**B,D**) substitutions. In (**A,B**) the ordinates show fN values, representing the fraction of the given site-type that experienced a C > T (G > A) mutation at least once. In (**C,D**) the ordinates show fN*, the fraction of the given site-type that experienced a C > T (G > A) mutation at least once, multiplied by the number of times the given mutation was observed. Note that in these figures we have separated codon pairs involving mutation of CpG of CpG-bearing codons (black columns) from those not involving mutation of CpG dinucleotide (light grey columns). In the panels separate horizontal lines indicate the average fN values (**A,B**) and fN* values (**C,D**) for the eight CpG bearing codons and for codons-pairs where the C > T and G > A mutation did not involve a CpG dinucleotide.

Our analyses of the mutation spectra of passenger genes (Supplementary files 1–4) have shown that the eight CpG bearing codons show fN values for C > T mutations (PG_CSS_fN: 0.44132 ± 0.05202) and G > A mutations (PG_CAS_fN: 0.42945 ± 0.08683) of their CpG sites that are significantly (p = 3.25541E−22 and p = 2.26514E−19, respectively) higher than those for C and G sites that are not part of CpG sequences (PG_RESTS_fN: 0.10014 ± 0.04917; PG_RESTA_fN: 0.09797 ± 0.04688); see Supplementary files 2 and 4). The marked difference between the two categories is also reflected by their fN* values (p = 1.43784E−22 and p = 3.68869E−19, respectively) (Supplementary files 2 and 4, Fig. 1 and Table 1).

**Table 1:** fN and fN* values of C > T mutations of passenger genes, tumor suppressor genes and oncogenes.

Passenger genes		
CSS	fN: 0.44132 ± 0.05202	fN*: 0.77608 ± 0.17898
CAS	fN: 0.42945 ± 0.08683	fN*: 0.71487 ± 0.18314
3rdC	fN: 0.37204 ± 0.08547	fN*: 0.61339 ± 0.21179
1stG	fN: 0.40242 ± 0.16338	fN*: 0.64084 ± 0.23557
RESTS	fN: 0.10014 ± 0.04917	fN*: 0.12419 ± 0.06657
RESTA	fN: 0.09797 ± 0.04688	fN*: 0.12419 ± 0.07395
Tumor suppressor genes		
CSS	fN: 0.58056 ± 0.15156	fN*: 2.85868 ± 3.38079
CAS	fN: 0.42734 ± 0.12529	fN*: 1.42465 ± 1.07986
3rdC	fN: 0.31223 ± 0.09200	fN*: 0.49391 ± 0.17117
1stG	fN: 0.38912 ± 0.16380	fN*: 0.83745 ± 0.46106
RESTS	fN: 0.14449 ± 0.09297	fN*: 0.23570 ± 0.24489
RESTA	fN: 0.12348 ± 0.06982	fN*: 0.21334 ± 0.17756
Oncogenes		
CSS	fN: 0.51861 ± 0.06308	fN*: 1.63340 ± 1.31114
CAS	fN: 0.45605 ± 0.13601	fN*: 2.30639 ± 3.67931
3rdC	fN: 0.50524 ± 0.13406	fN*: 0.83669 ± 0.33442
1stG	fN: 0.60368 ± 0.13808	fN*: 1.40289 ± 0.72271
RESTS	fN: 0.12391 ± 0.06582	fN*: 0.23931 ± 0.31303
RESTA	fN: 0.11569 ± 0.05015	fN*: 0.54039 ± 1.50149

*CSS* C > T substitution of CpG sites, sense strand, *CAS* C > T substitution of CpG sites, antisense strand, *3rdC* C > T substitution of CpG sites spanning codon boundaries, sense strand, *1stG* C > T substitution of CpG sites spanning codon boundaries, antisense strand, *RESTS* C > T substitution of sites that are not part of CpG dinucleotides, sense strand, *RESTA* C > T substitution of sites that are not part of CpG dinucleotides, antisense strand.

The most plausible explanation for this observation is that cytosine methylation of CpG sites significantly increases mutations in both the sense and antisense strands. In the case of selectively neutral passenger genes selection has no influence on somatic mutations. This conclusion has further support from our observation that although hypermutability of CpG sites of NCG codons (Thr, Pro, Ala and Ser) in the sense strand alter the amino acid, whereas mutations in the antisense strand are silent, the fN and fN* values for the C > T and G > A mutations of these codons are not significantly different (p = 0.18959) (see Supplementary files 2 and 4, Fig. 1).

The lack of a significant role of selection is also clear from our analysis of CpG sites spanning codon boundaries, i.e. when the third nucleotide of codon is C, whereas the first nucleotide of the next codon is G (categories 3rdC and 1stG). Mutations C > T affecting such CpG sites in the sense strand (categories 3rdC) are silent, whereas in the antisense strand (categories 1stG) result in missense mutations (Supplementary Table 2). As shown in Supplementary files 2 and 4 and Fig. 1, there is no significant difference (p = 0.51491) between the fN values of categories 3rdC (PG_3rdC_fN: 0.37204 ± 0.08547) and 1stG (PG_1stG_fN: 0.40242 ± 0.16338). The absence of a significant difference in the rates of silent and missense mutations at CpG sites spanning codon boundaries suggests that selection has no role in the elevated rate of mutation at these CpG sites.

Analysis of somatic C > T and G > A substitutions of CpG-bearing codons of individual passenger genes has revealed that the average fN values of C > T mutations (0.56015 ± 0.175710) and G > A mutations (0.54425 ± 0.22557) of their CpG sites show moderate variation (Supplementary file 3). Low recurrence rates of somatic mutations of passenger genes, reflected by relatively low fN* values of their C > T mutations (1.03932 ± 0.62458) and G > A mutations (0.87257 ± 0.38549) is in harmony with the notion that selection does not play a role in the case of recurrence of passenger mutations.

#### Tumor suppressor genes

**Figure 2:**
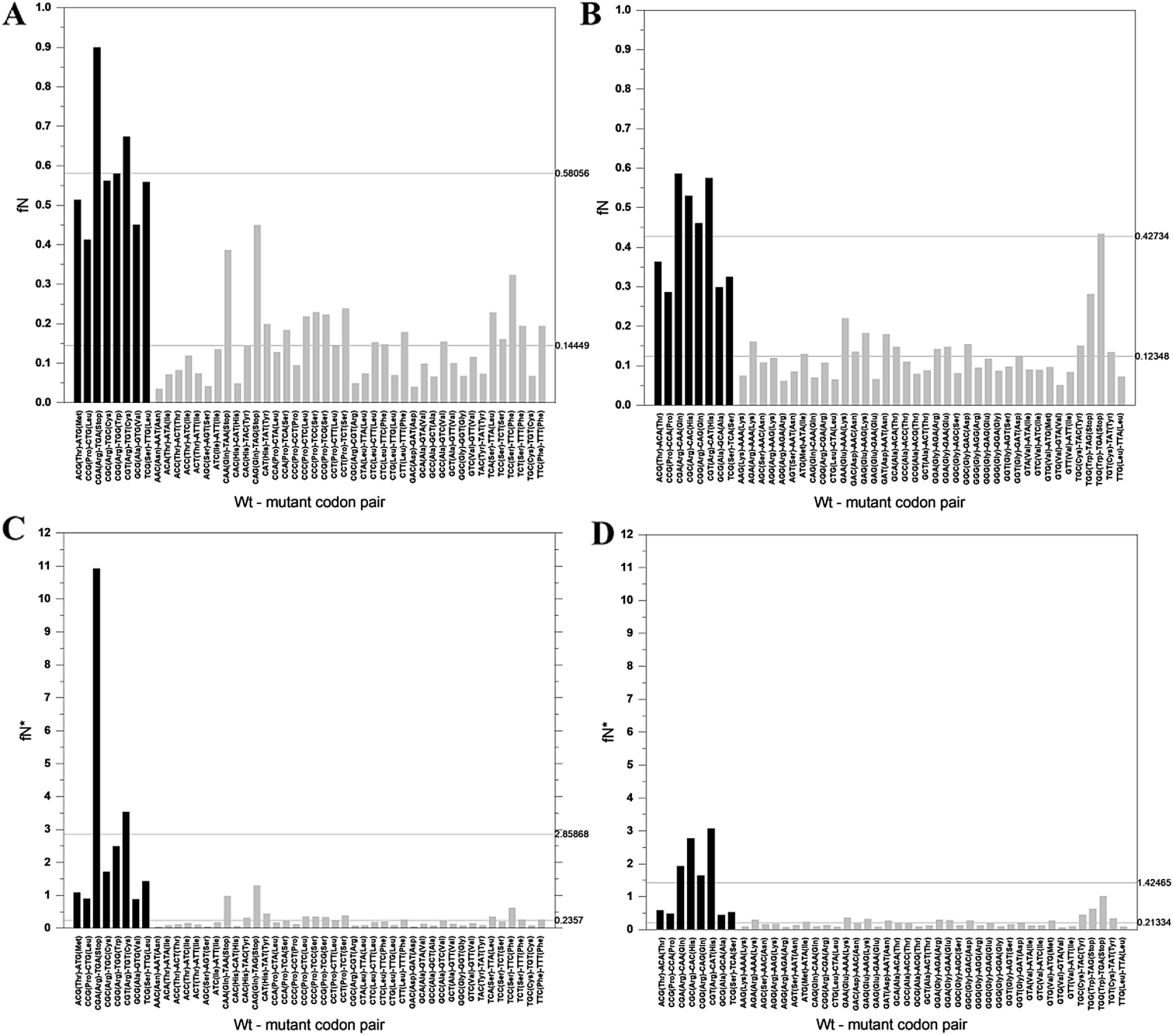
Frequency and recurrence of somatic C > T and G > A substitutions of tumor suppressor genes (TSGs). The abscissas list the wild type and mutant codon pairs resulting from C > T (**A,C**) or G > A (**B,D**) substitutions. In (**A,B**) the ordinates show fN values, representing the fraction of the given site-type that experienced a C > T (G > A) mutation at least once. In (**C,D**) the ordinates show fN*, the fraction of the given site-type that experienced a C > T (G > A) mutation at least once, multiplied by the number of times the given mutation was observed. Note that in these figures we have separated codon pairs involving mutation of CpG of CpG-bearing codons (black columns) from those not involving mutation of CpG dinucleotide (light grey columns). In the panels separate horizontal lines indicate the average fN values (**A,B**) and fN* values (**C,D**) for the eight CpG bearing codons and for codons-pairs where the C > T and G > A mutation did not involve a CpG dinucleotide.

In the case of the mutation spectra of tumor suppressor genes (Supplementary files 1–4) the fN values for C > T mutations (TSG_CSS_fN: 0.58056 ± 0.15156) and G > A mutations (TSG_CAS_fN: 0.42734 ± 0.125293) of CpG dinucleotides of CpG bearing codons were also significantly higher (p = 3.10198E−14 and p = 2.19404E−12 respectively) than those for C and G sites that are not part of CpG dinucleotides (TSG_RESTS_fN: 0.14449 ± 0.09297; TSG_RESTA_fN: 0.12348 ± 0.06982) (Supplementary files 2 and 4, Fig. 2 and Table 1).

Comparison of the fN values for C > T mutations and G > A mutations of CpG dinucleotides of CpG bearing codons of TSGs with those of PGs (Supplementary files 2 and 4), revealed that the TSG_CSS_fN values were significantly (p = 0.02764) higher than those of PG_CSS_fN, whereas no significant difference was observed in the case of the PG_CAS_fN versus TSG_CAS_fN comparison. Inspection of the data reveals that the marked difference between the fN values of PGs and TSGs—only in the sense strand—is due to increased rate of mutations of CGA > TGA (Arg > STOP) mutations in TSGs in the sense strand, but no increased rate of the CGA > CAA (Arg > Gln) mutation of the same CpG site in the antisense strand (cf. Figs. 1 and 2). This asymmetry of the mutations of the CpG site of the CGA codon in the sense and antisense strands reflects strong selection for truncating nonsense mutations of tumor suppressor genes over missense mutations. The dominance of the CGA > TGA (Arg > STOP) mutations in TSGs is even more obvious when recurrence of mutations is also taken into account: the fN* value of CGA > TGA mutations of TSGs is an order of magnitude higher than in the case of PGs (Supplementary file 2, Figs. 1 and 2).

Analysis of somatic C > T and G > A substitutions of CpG-bearing codons of individual tumor suppressor genes has revealed that the average fN values of C > T mutations (0.65829 ± 0.15826) and G > A mutations (0.53347 ± 0.15875) of their CpG sites show moderate variation (Supplementary file 3 ). The fact that the fN values of C > T mutations (0.65829 ± 0.15826) are significantly higher (p = 6.37314E-6) than those of G > A mutations (0.53347 ± 0.15875) reflects the dominance of CGA > TGA (Arg > STOP) mutations among driver mutations of TSGs.

The fN* values of individual TSGs display marked variations (4.97814 ± 16.38148 for C > T mutations, 2.63458 ± 10.35570 for G > A mutations), some TSGs showing extremely high fN* values, in harmony with the notion that selection plays a major role in the high rate of recurrence of driver mutations affecting CpG sites of these genes. We will illustrate and discuss this point on selected examples of TSGs with high fN* values in section “Significance of hypermutability of CpG dinucleotides in carcinogenesis”.

#### Oncogenes

**Figure 3:**
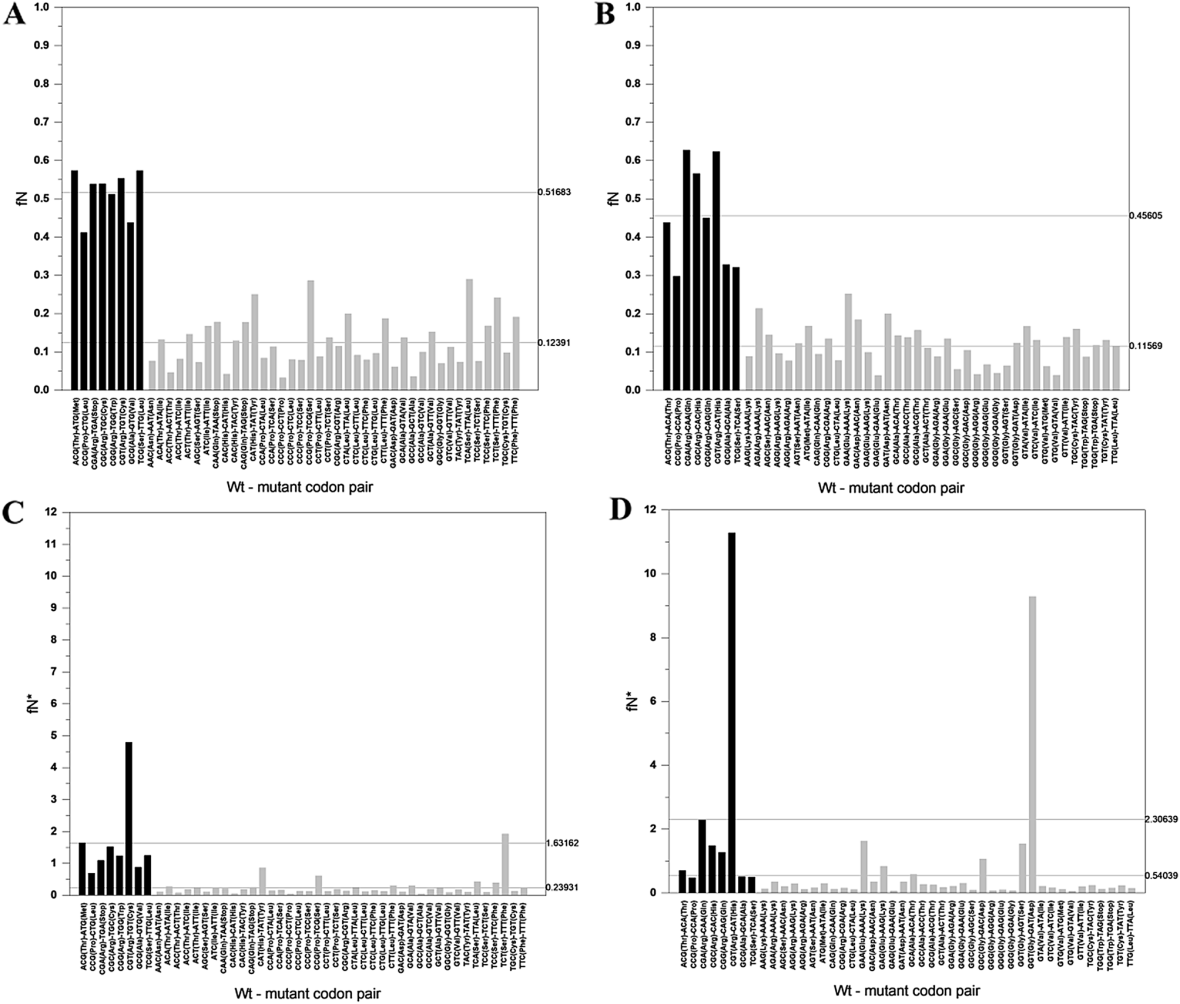
Frequency and recurrence of somatic C > T and G > A substitutions of proto-oncogenes (OGs). The abscissas list the wild type and mutant codon pairs resulting from C > T (**A,C**) or G > A (**B,D**) substitutions. In (**A,B**) the ordinates show fN values, representing the fraction of the given site-type that experienced a C > T (G > A) mutation at least once. In panels C and D the ordinates show fN*, the fraction of the given site-type that experienced a C > T (G > A) mutation at least once, multiplied by the number of times the given mutation was observed. Note that in these figures we have separated codon pairs involving mutation of CpG of CpG-bearing codons (black columns) from those not involving mutation of CpG dinucleotide (light grey columns). In the panels separate horizontal lines indicate the average fN values (**A,B**) and fN* values (**C,D**) for the eight CpG bearing codons and for codons-pairs where the C > T and G > A mutation did not involve a CpG dinucleotide.

In the case of the mutation spectra of proto-oncogenes (Supplementary files 1–4) the fN values for C > T mutations and G > A mutations of CpG dinucleotides of CpG bearing codons were also significantly higher (OG_CSS_fN versus OG_RESTS_fN: p = 5.69277E−20; OG_CAS_fN versus OG_RESTA_fN: p = 7.73026E−16) than those for C and G sites that are not part of CpG dinucleotides (Supplementary files 2 and 4, Fig. 3 and Table 1).

The fN* values of oncogenes, reflecting recurrence of C > T (G > A) mutations of the different CpG-bearing codons (CGN and NCG) showed greater variation than in the case of passenger genes, indicating positive selection for missense mutations. Of particular interest are the CGT > TGT (Arg > Cys) and CGT > CAT (Arg > His) mutations of oncogenes that have fN* values an order of magnitude higher than in the case of PGs.

Analyses of somatic C > T and G > A substitutions of CpG-bearing codons of individual proto-oncogenes has revealed that the fN values of C > T mutations (0.60967 ± 0.16834) and G > A mutations (0.56989 ± 0.18454) of their CpG sites show moderate variation (Supplementary file 3). The fact that the fN values of C > T mutations are not significantly different (p = 0.24455) from those of G > A mutations reflects the dominance of missense mutations affecting CGN codons.

The fN* values of CpG bearing codons of individual OGs display marked variations (1.84007 ± 2.25446 for C > T mutations, 2.97465 ± 10.81006 for G > A mutations). Some OGs show extremely high fN* values, in harmony with the notion that selection plays a major role in the high rate of recurrence of driver mutations affecting CpG sites of these genes. We will illustrate and discuss this point on selected examples of OGs with high fN* values in section “Significance of hypermutability of CpG dinucleotides in carcinogenesis”.

Comparison of the frequency and recurrence of somatic C > T and G > A substitutions of CpG bearing codons of passenger genes with those of tumor suppressor genes and oncogenes (Table 1) thus suggests that driver mutations of both oncogenes and tumors suppressor genes benefit from hypermutability of CpG bearing codons. In the case of tumor suppressor genes it is the hypermutability of the CGA codon for arginine that contributes to carcinogenesis, since it converts it to a stop codon, explaining why CGA > TGA mutation has exceptionally high fN* values in the case of tumor suppressor genes (Supplementary file 2, Fig. 2). In the case of oncogenes it is the hypermutability of key CGN codons that contributes most significantly to carcinogenesis, since—with the exception of the CGA > TGA mutation—all C > T and G > A substitutions of these codons result in missense mutations.

Our analyses thus suggest that methylation and hypermutability of CGN codons of arginine play an important role in carcinogenesis as it facilitates the generation of driver mutations that activate proto-oncogenes or inactivate tumor suppressor genes. In the following section we discuss the mutation patterns of some tumor suppressor genes and oncogenes to illustrate the significance of CpG hypermutability in carcinogenesis.

### Significance of hypermutability of CpG dinucleotides in carcinogenesis

#### Significance of hypermutability of CpG dinucleotides of tumor suppressor genes

Driver mutations of tumor suppressor genes frequently truncate the tumor suppressor proteins therefore the heuristic approach of Vogelstein et al.^8^ classifies driver genes as tumor suppressor genes if > 20% of the recorded mutations in the gene are truncating (nonsense or frameshift) mutations.

Our earlier analyses of human mutation data revealed that out of the eighteen sense codons that can give rise to a nonsense codon by single base substitution, the CGA codon is exceptional: it gives rise to the TGA stop codon at an order of magnitude higher rate than the other stopogenic codons^5^. In view of the hypermutability of the CpG of the CGA codon it is not surprising that it contributes significantly to the inactivation of tumor suppressor genes and thus, to carcinogenesis.

Inspection of the data on individual tumor suppressor genes (Supplementary file 3) reveals that most cases with exceptionally high fN* values of C > T mutations of CpG dinucleotides reflect CGA > TGA mutations at multiple sites, resulting in Arg > Stop mutations.

For example, the tumor suppressor gene APC has numerous CGA (Arg) codons that are recurrently (> 50) mutated to TGA (Stop) codons: R213*, R216*, R232*, R283*, R302*, R499*, R554*, R564*, R805*, R876*, R1114*, R1450*, see Supplementary file 1. It is noteworthy that these codons of APC show high rates of recurrent mutations of the CpG site only in the sense strand, reflecting positive selection for nonsense mutations over missense mutation. This bias also explains why fN*_CSS value of APC (9.10831) is several fold higher than the fN*_CAS value (1.36865); see Supplementary file 3.

A similar situation holds for the cyclin-dependent kinase inhibitor gene CDKN2A. This tumor suppressor gene is inactivated most frequently by recurrent nonsense mutations R80* and R58*, resulting from hypermutability of CpG sites of the CGA codons of these arginines. High rates of recurrent mutations of the CpG sites are observed only in the sense strand (Supplementary file 1), reflecting positive selection for nonsense mutations over missense mutations. This bias also explains why fN*_CSS of CDKN2A (22.74679) is several fold higher than its fN*_CAS value (1.52976); see Supplementary file 3.

The tumor suppressor gene, PIK3R1, encoding phosphoinositide-3-kinase, has several CGA (Arg) codons; recurrent mutations of R348*, R358*, R386*,R461*, R534*, R557*, R631*, R639* and R642* (Supplementary file 1), contribute significantly to truncation of this suppressor, thereby promoting carcinogenesis. 

In the case of several tumor suppressor genes both recurrent nonsense and missense mutations of CGN codons can lead to the inactivation of the genes. For example, recurrent R196*, R213*, R306*, R342*, R175H, R248W, R248Q, R273C, R273H and R282W mutations play a key role in the inactivation of the TP53 gene. The contribution of both nonsense and missense mutations of CGN codons to inactivation of TP53 is reflected in the fact that both the fN*_CSS (134.43194) and fN*_CAS of TP53 (85.63194) are extremely high (see Supplementary file 3). Similarly, driver mutations of the phosphatase and tensin homolog gene, PTEN include recurrent missense and nonsense mutations R130G, R130Q, R130*, R233*, R173C^10^, explaining why both the fN*_CSS (30.23333) and fN*_CAS of PTEN (20.00000) are high (Supplementary file 3). 

#### Significance of methylation and hypermutability of CpG dinucleotides of oncogenes

Most known oncogenes were found to be recurrently mutated at the same amino acid positions, therefore the heuristic approach of Vogelstein et al.^8^ classifies driver genes as oncogenes if > 20% of the recorded mutations in the gene are at recurrent positions and are missense.

A survey of the mutation data on proto-oncogenes (Supplementary file 3) identified several cases where exceptionally high rates of recurrent driver mutations involved C > T and G > A mutations of CpG dinucleotides of CpG bearing codons. These oncogenes had very high fN* values in both the sense and antisense strands; as examples we may mention IDH1 (fN*_CSS: 14.92000; fN*_CAS: 79.92500), GNAS (fN*_CSS: 8.44775; fN*_CAS: 7.72019), PIK3CA (fN*_CSS: 5.77961; fN*_CAS: 8.46327) and NFE2L2 (fN*_CSS: 2.66667: fN*_CAS: 8.75000).

A particularly instructive example is the case of the isocitrate dehydrogenase genes, IDH1 and IDH2. These oncogenes are affected by recurrent driver mutations in tumors^11,12^. Somatic missense mutations of Arg-132 of IDH1 and its equivalent, Arg-172 in IDH2 result in the loss of normal isocitrate dehydrogenase activity of the enzymes and lead to the abnormal production of 2-hydroxyglutarate. Arg-132 of IDH1 and Arg-172 of IDH2 are located in the enzymes’ active sites, suggesting a direct impact of the mutations on their catalytic properties. 2-Hydroxyglutarate inhibits many alpha-ketoglutarate-dependent enzymes, including histone and DNA demethylases, causing widespread epigenetic changes in the genome thereby promoting tumorigenesis.

Arg-132 of IDH1 and Arg-172 of IDH2, affected by recurrent mutations, however, differ in codon usage. In the case of IDH1 the conserved Arg-132 is encoded by CGT, a codon containing CpG, whereas the equivalent Arg (Arg-172) of IDH2 is encoded by AGG. As shown in Supplementary file 1, the majority of substitutions of Arg-132 of IDH1 convert CGT to CAT (His, 1564 counts), TGT (Cys, 343 counts) reflecting hypermutability of hypermethylated sense and antisense CpG dinucleotides of the codon. The remaining minority of substitutions of IDH1 replaces Arg-132 by Ser (58 counts), by Gly (81 counts) and by Leu (51 counts), through mutations that do not involve hypermutability of CpG dinucleotides. Inspection of the substitutions of Arg-172 of IDH2 (Supplementary file 1) reveals that recurrent single base substitutions of the AGG codon of Arg-172 replace the amino acid by Gly (18 counts), Trp (15 counts), Lys (90 counts), Thr (3 counts), Met (16 counts) or Ser (29 counts). Thus, comparison of the pattern of driver substitutions of the two isocitrate dehydrogenase genes makes it clear that although a wide variety of missense substitutions of these active site arginines are oncogenic, the hypermutability of the CpG dinucleotide is responsible for the higher frequency of substitutions at the IDH1 site than at the IDH2 site as well as for the dominance of Arg > Cys and Arg > His substitutions over other types of missense mutations of IDH1.

Recurrent mutations of Arg 844 of the oncogene, GNAS, result in its constitutive activation and leads to the autonomous synthesis of cyclic adenosine monophosphate^13–15^. Although replacement of Arg-844 by virtually any amino acid results in constitutive activation of GNAS^13^, the majority of mutations of this arginine codon (CGC) convert it to Cys (TGC, 337 counts) or His (CAC, 297 counts), with a small fraction of recurrent substitutions replacing Arg by Ser residue (18 counts) (see Supplementary file 1). Clearly, it is the hypermutability of the CpG dinucleotide that renders this codon vulnerable and favors Arg > Cys and Arg > His substitutions over other types of missense mutations.

Recurrent missense somatic mutations of the phosphoinositide-3-kinase gene, PIK3CA include R88Q, E542K, E545K, E545A, Q546K, H1047R and H1047L. The H1047R mutation is the most frequent. Mutations E542K, E545K, and H1047R induce an oncogenic transformation with high efficiency, but the role of the R88Q (CGA > CAA) mutation is more controversial. It has been shown that the R88Q mutant PIK3CA did not promote tumorigenesis more than wild type PIK3CA in vivo, suggesting that it may be a passenger mutation^16^. Thus, in this case recurrence of the R88Q mutation has more to do with hypermutability than with selection.

Several recurrent missense mutations of the nuclear factor (erythroid-derived 2)-like 2 gene, NFE2L2 have been identified, mutations of the R34 position being the most frequent^17^. A variety of recurrent mutations of R34 (CGA) have been shown to drive constitutive NFE2L2 activation: R34Q (CGA > CAA, count 28), R34G (CGA > GGA, count 35), R34P (CGA > CCA, count 20), R34L (CGA > CTA, count 4). The fact that the R34G (CGA > GGA) mutation is more frequent than the R34Q (CGA > CAA) mutation indicates that selection favoring R34G mutation overrides the effect of hypermutability that would favor the R34Q (CGA > CAA) mutation.

There are also some cases where oncogenic driver mutations arise as a result of mutation of CpG dinucleotides of codons of the NCG group of CpG bearing codons. For example, the ACG codon encoding Thr790 of EGFR kinase is recurrently (count 140) altered by ACG > ATG mutation (Supplementary file 1). It has been shown that the T790M mutation increases the ATP affinity of the kinase reducing the potency of ATP-competitive kinase inhibitors^18^. The fact that mutations of Thr790 of EGFR kinase did not include mutations of the CpG in the antisense strand, suggests that recurrence of the T790M mutation is due primarily to positive selection for missense mutation.

### Vulnerability of *cancer* genes bearing CGN codons

The data discussed in the previous sections suggest that hypermutability of CpG dinucleotides of the four CGN codons for arginine may serve as Achilles' heels of cancer genes. Our analyses have also revealed that there are marked differences in the CpG-dependent vulnerability of various tumor suppressor genes and various oncogenes: TSGs appear to be more vulnerable than OGs. This difference is due to the fact that in the case of OGs there are important restrictions: to serve as sites of driver mutations hypermutable CpG bearing codons must occupy positions critical for the activity of the OG and the mutation must alter (but not truncate) the proto-oncogene. In this respect, CpG bearing codons of TSGs have a much greater freedom of choice to impair or eliminate the activity of the TSG by missense, and particularly by nonsense mutations. This is especially true for CGA codons, as C > T mutations leading to TGA stop codons will truncate and inactivate the protein, almost independent of their position in the protein sequence. The dominant role of CGA hypermutability in the sense strand is illustrated by the fact that in the case of TSGs fN values for the sense strand are higher than those for the antisense strand (see section “Significance of hypermutability of CpG dinucleotides of tumor suppressor genes”).

As discussed in section “Significance of hypermutability of CpG dinucleotides in carcinogenesis”, even within the group of tumor suppressor genes, there are significant differences in sensitivity to CpG-dependent accumulation of driver mutations, as reflected by their fN*_CSS and fN*_CAS values (Supplementary file 3). According to these criteria, the most vulnerable TSGs include TP53, PTEN, CDKN2A, FBXW7, APC and PIK3R1. As for OGs, the most vulnerable genes with highest fN*_CSS and fN*_CAS values include IDH1, GNAS, PIK3CA and NFE2L2 (see section “Significance of methylation and hypermutability of CpG dinucleotides of oncogenes”).

In view of the exceptional vulnerability of some tumor suppressor genes, it may be expected that there may be strong evolutionary pressure to prevent loss of these genes in animals with long lifespan. It is noteworthy in this respect that TP53 copy number expansion in elephants has been shown to be associated with the evolution of increased body size and long lifespan through reinforcing the anti-cancer mechanisms of the major ‘guardian of the genome’ TP53^19^. Similarly, recent studies have shown that high duplication levels of the tumor suppressor PIK3R1 in two ageing extremists, the naked mole rat and the greater mouse-eared bat resulted from convergent duplication events, whereby coding sequences of this tumor suppressor were independently duplicated multiple times in both of these long-lived species^20^.

## Age dependent changes in the vulnerability of *cancer* genes bearing CGN codons

Genomic 5-methyldeoxycytidine levels have been shown to decline with in vivo age so that the probability of the loss of 5mC from a specific CpG sequence or site increases with time^21,22^. In view of the key role of the methylation of CpG sites in somatic mutations it is to be expected that genome-wide, age dependent loss of CpG methylation is also reflected in changes in the rate and spectra of somatic mutations. In harmony with this expectation, data from various murine and human tissues demonstrate that the majority of somatic mutations accumulate early in life before full body maturation; the rate of accumulation significantly slows down thereafter^22–24^. This slow down is also associated with a change in mutation spectra; whereas at young age the mutation spectra consist of G-C to A-T transitions (primarily at CpG sites), at old age, other patterns of mutations become dominant^25^.

As pointed out by Rozhok and DeGregori^24^, there is an apparent contradiction between the observations that mutation accumulation occurs largely during development phase, whereas cancer incidence increases exponentially with age. In a seminal paper Armitage and Doll^26^ proposed the multi-stage theory of carcinogenesis stating that carcinogenesis typically requires 6–7 mutations and/or other cell alteration to malignantly transform a cell, based on the evidence that the age-dependent exponential increase in cancer incidence follows mathematically the 6th power of age. In harmony with this multi-stage theory of carcinogenesis we suggest that the majority of the pro-carcinogenic driver mutations that arise in the development phase affect CpG sites, such as those occurring CGN codons, setting the stage for the accumulation of the critical number of additional driver mutations that will be manifested as cancer incidence.

This explanation has an important implication: the carcinogenetic process is most likely to be initiated by CpG dependent driver mutations of cancer genes that are most sensitive, most vulnerable to such mutations. It is noteworthy in this respect that analyses of the temporal order of driver genes' mutations across clinical stages in different cancer types have shown that mutations of TP53 were the critical initiation events in the majority of cancer types^27,28^. Furthermore, the list of typically early drivers includes most other highly recurrent CpG-dependent cancer driver genes identified in the present work, such as TP53, PIK3CA, CDKN2A, PTEN, APC, RB1, FBXW7, NFE2L2, IDH1, PIK3R1^28^, consistent with the notion that hypermutability of CpG of these cancer genes plays a critical role in the initiation of carcinogenesis.

## Conclusions

The present work was motivated by recent studies that have revealed significant prevalence of arginine mutations in various cancer types, an observation that was usually interpreted as a reflection of the critical roles of this amino acid in the function of the proteins encoded by cancer genes^1–4^.

However, high rates of mutations of arginine residues might also be due to increased mutability of arginine codons as the four CGN codons of this amino acid contain CpG dinucleotides that may be methylated and may thus experience elevated rates of C > T (G > A) mutations at these sites. To assess the contributions of CpG hypermutability and selection to arginine mutations we have first analyzed the C > T (G > A) mutation patterns of passenger genes, i.e. selectively neutral genes that do not play a role in carcinogenesis^7^. The results of these analyses have confirmed that CpG hypermutability of CGN codons results in a marked increase in mutation probability at these sites as compared to C and G sites that are not part of CpG sequences, even in the absence of selection (Fig. 1).

Analyzes of the C > T (G > A) mutation patterns of cancer genes (tumor suppressor genes, proto-oncogenes) that are positively selected for mutations altering amino acids^7^ revealed that these mutations are also driven primarily by CpG hypermutability (Figs. 2 and 3). The actual mutation patterns at CpG sites, however, are modulated by selection in as much as it enhances or diminishes mutations rates. Comparison of the mutation patterns of CGN codons of passenger genes (Fig. 1) and cancer genes (Figs. 2 and 3) revealed that in the case of tumor suppressor genes there is significant selection for nonsense mutations (CGA > TGA; Arg > Stop) that inactivate the genes (Fig. 2), whereas in the case of proto-oncogenes there is strong selection for missense mutations (e.g. CGT > TGT, Arg > Cys; CGT > CAT, Arg > His) that activate these genes (Fig. 3).

Our results thus suggest that, due to methylation and hypermutability of CGN codons of arginines, these arginine codons may serve as Achilles' heels of cancer genes as they may increase their vulnerability. Our analyses have also shown that, depending on their CGN content and codon usage, there are marked differences in the vulnerability of various tumor suppressor genes and various oncogenes (see section “Vulnerability of cancer genes, bearing CGN codons”). The significance of this point may be best illustrated by the case of the two closely related oncogenes, IDH1 and IDH2. As discussed in section “Significance of methylation and hypermutability of CpG dinucleotides of oncogenes”, somatic missense mutations affecting an equivalent Arg residue at the active sites of these isocitrate dehydrogenase genes serve as driver mutations in tumors. IDH1 and IDH2, however, differ in codon usage: in IDH1 the active site Arg is encoded by CGT, a codon containing a hypermutable CpG dinucleotide, whereas the equivalent Arg of IDH2 is encoded by AGG that does not contain a CpG sequence. This difference is reflected in a marked difference in the susceptibility of the two proto-oncogenes to acquire driver mutations: due to hypermutability of the CpG dinucleotide the fN*_CSS and fN*_CAS values of IDH1 are significantly higher than those of IDH2 (Supplementary file 3).

Based on their high fN*_CSS and fN*_CAS values (Supplementary file 3), the most vulnerable cancer genes include the tumor suppressors TP53, PTEN, CDKN2A, FBXW7, APC and PIK3R1, as well as proto-oncogenes IDH1, GNAS, PIK3CA and NFE2L2. Differences in the susceptibility of cancer genes to acquire driver mutations have important implications for the carcinogenetic process. Carcinogenesis is most likely to be initiated by CpG dependent driver mutations of cancer genes that are most susceptible to such mutations. In harmony with this prediction, the list of typically early drivers includes most of the CpG-dependent cancer driver genes identified in the present work, such as TP53, PIK3CA, CDKN2A, PTEN, APC, RB1, FBXW7, NFE2L2, IDH1, PIK3R1^28^.

We have pointed out that, in view of the vulnerability and critical roles of the cancer genes listed above, in animals with long lifespan there might be evolutionary pressure to protect these tumor suppressor genes from inactivation (proto-oncogenes from activation) to ensure cancer resistance. As shown by several recent studies, one of the major evolutionary mechanisms exploited by long-lived animals is duplication of tumor suppressor genes^29,30^. It is noteworthy that TP53, the most critical tumor suppressor gene, is represented by multiple copies in elephants, reinforcing the anti-cancer function of the gene^19^. The significance of TP53 copy number expansion is underlined by the observation that it has been associated with the evolution of long lifespan in the lineage of elephants^19^. Further support for such protection of the most critical tumor suppressor genes came from recent studies on naked mole rat and the greater mouse-eared bat, animals with extremely long lifespan. The tumor suppressor PIK3R1 has been duplicated independently several times in both of these long-lived species^20^. Future studies might reveal whether high levels of duplication of the most susceptible tumor suppressor genes are a general evolutionary mechanism that is exploited by long-lived species to acquire cancer resistance.

Given the dominant nature of driver mutations that activate proto-oncogenes, copy number expansion of these genes is less likely to ensure a protective, evolutionary anticancer mechanism. However, during evolution of cancer resistant species proto-oncogenes activated through hypermutability of CpG dinucleotides might be under selective pressure to replace the vulnerable CpG bearing codons. Similarly, one might argue that evolution of cancer resistance may favor the replacement of CGA codons of tumor suppressor genes, rendering them less susceptible to inactivation. Studies addressing these questions are in progress in our lab, since—as pointed by Seluanov and coworkers^31^, understanding the molecular mechanisms of anticancer adaptations that evolved in different species is of critical importance as it may lead to new avenues in cancer treatment and prevention.

## Materials and methods

### Lists of genes analyzed

We have analyzed three different groups of human protein-coding genes, known to differ in selection for mutations in cancer^7^. As the gold standard of ’known’ cancer genes we have used the lists of oncogenes (OGs) and tumor suppressor genes (TSGs) identified by Vogelstein et al.^8^. As a control group, we have used the list of passenger genes (PGs) characterized by Bányai et al.^9^.

### Somatic mutation data

Cancer somatic mutation data were extracted from COSMIC v96 (COSMIC release v96, 31st May 2022), the Catalogue Of Somatic Mutations In Cancer (https://cancer.sanger.ac.uk/cosmic/download) which includes single nucleotide substitutions from targeted and genome-wide screens, affecting the coding sequence of human genes. Only confirmed somatic, point mutations that arose during tumor evolution were included in our analyses. Accordingly, for all subsequent analyses we have retained only transcripts containing substitutions that were annotated under ‘Mutation somatic status’ as Confirmed Somatic, i.e. confirmed to be somatic in the experiment by sequencing both the tumor and a matched normal tissue from the same patient. As to ’Sample Type, Tumor origin’: we have excluded mutation data from cell-lines, organoid-cultures and xenografts since they do not properly represent human tumor evolution at the organism level. Finally, we have removed redundant data (due to multiple deposition of the same mutation from the same experiment) so that each unique substitution was represented only once in the dataset used in our analyses. In the present work somatic mutations were combined from all cancer types.

Although the COSMIC files provide information on the nature of the nucleotide substitution, its position in the coding sequence and the effect of the substitution on the amino acid sequence of the protein, in most cases the reconstruction of the events leading to the substitution requires the identification of the wild type codon and the position of the substitution within the codon. To solve this problem, we have downloaded the files (All_COSMIC_Genes.fasta.gz) containing the nucleotide and amino acid sequences of the genes and—using the MUTATION_CDS information—have identified the sequences of the wild type sense codons and the mutant stop codons (see Supplementary file 1).

### Substitution metrics

To analyze the impact of CpG methylation on mutation spectra we have compared the relative frequency of C > T (G > A) substitutions of nucleotides present in CpG dinucleotides with those of C (G) nucleotides that are not part of CpG dinucleotides. To facilitate these calculations, we have treated CpG bearing codons CGN (Arg) and NCG (Thr, Pro, Ala, Ser) separately from codons lacking CpG dinucleotides. However, CpG dinucleotides may also span the boundaries of neighboring codons (in the sense or antisense strand), i.e. if the third nucleotide of codon X is C, whereas the first nucleotide of the next codon (X + 1) is G. To check the influence of such “trans-codon CpGs” on C > T and G > A mutation spectra, we have analyzed the mutations of the affected sites as a separate group.

For each type of C (G) site of the various codons we have determined the fraction of sites (fN) that experienced a C > T (G > A) mutation at least once. This value was multiplied by the number of times the mutation was observed to calculate fN*, the parameter reflecting recurrence of that substitution.

### Statistical analyses

The statistical package of Origin 2018 was used for all data processing and statistical analysis. We report details of statistical tests in the Supplementary files of the respective sections. Statistical significance was set as a p value of < 0.05.

### Supplementary Information

Supplementary Tables.

Supplementary Information 1.

Supplementary Information 2.

Supplementary Information 3.

Supplementary Information 4.

## Data Availability

All data generated or analyzed during this study are included in this published article [and its supplementary information files].
