# Supplementary material for: Arginines of the CGN codon family are Achilles’ heels of cancer genes
Source: Sci Rep. 2024 May 22;14:11715. doi: 10.1038/s41598-024-62553-7 (PMC11111792; doi:10.1038/s41598-024-62553-7)
Supplement: Supplementary file 1 — Supplementary Tables. [file 41598_2024_62553_MOESM1_ESM.pdf]

Supplementary Table 1

Amino acid substitutions resulting from hypermutability of methylated CpG dinucleotides of codons containing CG sequences

Abbreviations:
WTC, Wild Type Codon; WTAA, Wild Type Amino Acid; WTN, Wild Type Nucleotide; MN, Mutant Nucleotide; MC, mutant codon; MAA, mutant amino acid; Category of mutation: NON, nonsense; MIS, missense; SIL, silent);

A) C>T substitutions of CpG sites in sense strand (CSS)

WTC	WTAA	WTN	MN	MC	MAA	Category
ACG	Thr	C	T	ATG	Met	MIS
CCG	Pro	C	T	CTG	Leu	MIS
CGA	Arg	C	T	TGA	STOP	NON
CGC	Arg	C	T	TGC	Cys	MIS
CGG	Arg	C	T	TGG	Trp	MIS
CGT	Arg	C	T	TGT	Cys	MIS
GCG	Ala	C	T	GTG	Val	MIS
TCG	Ser	C	T	TTG	Leu	MIS

B) G>A substitutions of CpG sites in antisense strand (CAS)

WTC	WTAA	WTN	MN	MC	MAA	Category
ACG	Thr	G	A	ACA	Thr	SIL
CCG	Pro	G	A	CCA	Pro	SIL
CGA	Arg	G	A	CAA	Gln	MIS
CGC	Arg	G	A	CAC	His	MIS
CGG	Arg	G	A	CAG	Gln	MIS
CGT	Arg	G	A	CAT	His	MIS
GCG	Ala	G	A	GCA	Ala	SIL
TCG	Ser	G	A	TCA	Ser	SIL

Supplementary Table 2

Amino acid substitutions resulting from hypermutability of methylated CpG dinucleotides of codons containing CG sequences spanning codon boundaries

Abbreviations: WTC, Wild Type Codon; WTAA, Wild Type Amino Acid; WTN, Wild Type Nucleotide; MN, Mutant Nucleotide; MC, mutant codon; MAA, mutant amino acid; Category of mutation: NON, nonsense; MIS, missense; SIL, silent);

C) C>T substitutions of CpG sites in sense strand (3rdC)

WTC	WTAA	WTN	MN	MC	MAA	Category
AAC	Asn	C	T	AAT	Asn	SIL
ACC	Thr	C	T	ACT	Thr	SIL
AGC	Ser	C	T	AGT	Ser	SIL
ATC	Ile	C	T	ATT	Ile	SIL
CAC	His	C	T	CAT	His	SIL
CCC	Pro	C	T	CCT	Pro	SIL
CGC	Arg	C	T	CGT	Arg	SIL
CTC	Leu	C	T	CTT	Leu	SIL
GAC	Asp	C	T	GAT	Asp	SIL
GCC	Ala	C	T	GCT	Ala	SIL
GGC	Gly	C	T	GGT	Gly	SIL
GTC	Val	C	T	GTT	Val	SIL
TAC	Tyr	C	T	TAT	Tyr	SIL
TCC	Ser	C	T	TCT	Ser	SIL
TGC	Cys	C	T	TGT	Cys	SIL
TTC	Phe	C	T	TTT	Phe	SIL

D) G>A substitutions of CpG sites in antisense strand (1stG)

WTC	WTAA	WTN	MN	MC	MAA	Category
GAA	Glu	G	A	AAA	Lys	MIS
GAC	Asp	G	A	AAC	Asn	MIS
GAG	Glu	G	A	AAG	Lys	MIS
GAT	Asp	G	A	AAT	Asn	MIS
GCA	Ala	G	A	ACA	Thr	MIS
GCC	Ala	G	A	ACC	Thr	MIS
GCG	Ala	G	A	ACG	Thr	MIS
GCT	Ala	G	A	ACT	Thr	MIS
GGA	Gly	G	A	AGA	Arg	MIS
GGC	Gly	G	A	AGC	Ser	MIS
GGG	Gly	G	A	AGG	Arg	MIS
GGT	Gly	G	A	AGT	Ser	MIS
GTA	Val	G	A	ATA	Ile	MIS
GTC	Val	G	A	ATC	Ile	MIS
GTG	Val	G	A	ATG	Met	MIS
GTT	Val	G	A	ATT	Ile	MIS
